# Gastrointestinal bleeding with Klippel–Trenaunay syndrome: a case report

**DOI:** 10.1186/s12876-021-01891-6

**Published:** 2021-08-05

**Authors:** Lin Han, Shifeng Chen, Shengping Jiang

**Affiliations:** 1 Department of Rehabilitation Medicine, The Sixth Affiliated Hospital of Guangxi Medical University: The First People’s Hospital of Yulin, No. 495 Jiaoyu Road, Yulin, 537000 China; 2 Department of Pediatrics, The Sixth Affiliated Hospital of Guangxi Medical University: The First People’s Hospital of Yulin, No. 495 Jiaoyu Road, Yulin, 537000 China; 3 Department of Radiology, The Sixth Affiliated Hospital of Guangxi Medical University: The First People’s Hospital of Yulin, No. 495 Jiaoyu Road, Yulin, 537000 China

**Keywords:** Klippel–Trenaunay syndrome, Gastrointestinal tract, Gastrointestinal hemorrhage, Case report

## Abstract

**Background:**

Gastrointestinal bleeding caused by gastrointestinal tract involvement in patients with Klippel–Trenaunay syndrome (KTS) is extremely rare and often overlooked.

Case presentation

A 9-year-old girl who presented with chronic gastrointestinal bleeding was admitted to our hospital. Laboratory examinations revealed microcytic hypochromic anemia and a positive fecal occult blood test. Computed tomography (CT) of the lower limbs combined with physical examination confirmed KTS. The pelvic CT showed concentric thickening of the sigmoid colon and rectum, with progressive enhancement after the administration of a contrast agent. Colonoscopy demonstrated vascular malformations of the sigmoid colon and rectum. The patient was finally diagnosed with KTS with gastrointestinal tract involvement. The patient improved after receiving conservative treatment.

**Conclusions:**

A suspicion of gastrointestinal tract involvement as an etiology for gastrointestinal bleeding should not be overlooked in patients with KTS. Endoscopy and imaging modalities can synergistically help diagnose this condition.

## Background

Klippel–Trenaunay syndrome (KTS) is a congenital and rare vascular malformation disorder that mainly involves the lower limbs. It is characterized by capillary, lymphatic and/or venous malformations and overgrowth of soft tissue and/or bone [1]. Gastrointestinal tract involvement in patients with KTS is uncommon, with associated bleeding accounting for approximately 1% of KTS cases according to a previous report [2]. Herein we report the case of a patient with rare gastrointestinal bleeding who underwent computed tomography (CT) and endoscopic examination.

## Case presentation

A 9-year-old girl with an 8-year history of chronic gastrointestinal bleeding was admitted to our hospital. The patient had no corresponding family history. Sclerotherapy in the varicose veins of her right lower limb was previously performed in another hospital. Physical examination showed a hypertrophic and longer right lower limb with vascular nevus. Blood was not observed during the digital rectal examination. Laboratory examinations revealed microcytic hypochromic anemia with a hemoglobin level of 7.3 g/dL (normal range; 11.0–15.0 g/dL) and a positive fecal occult blood test.

**Fig. 1:**
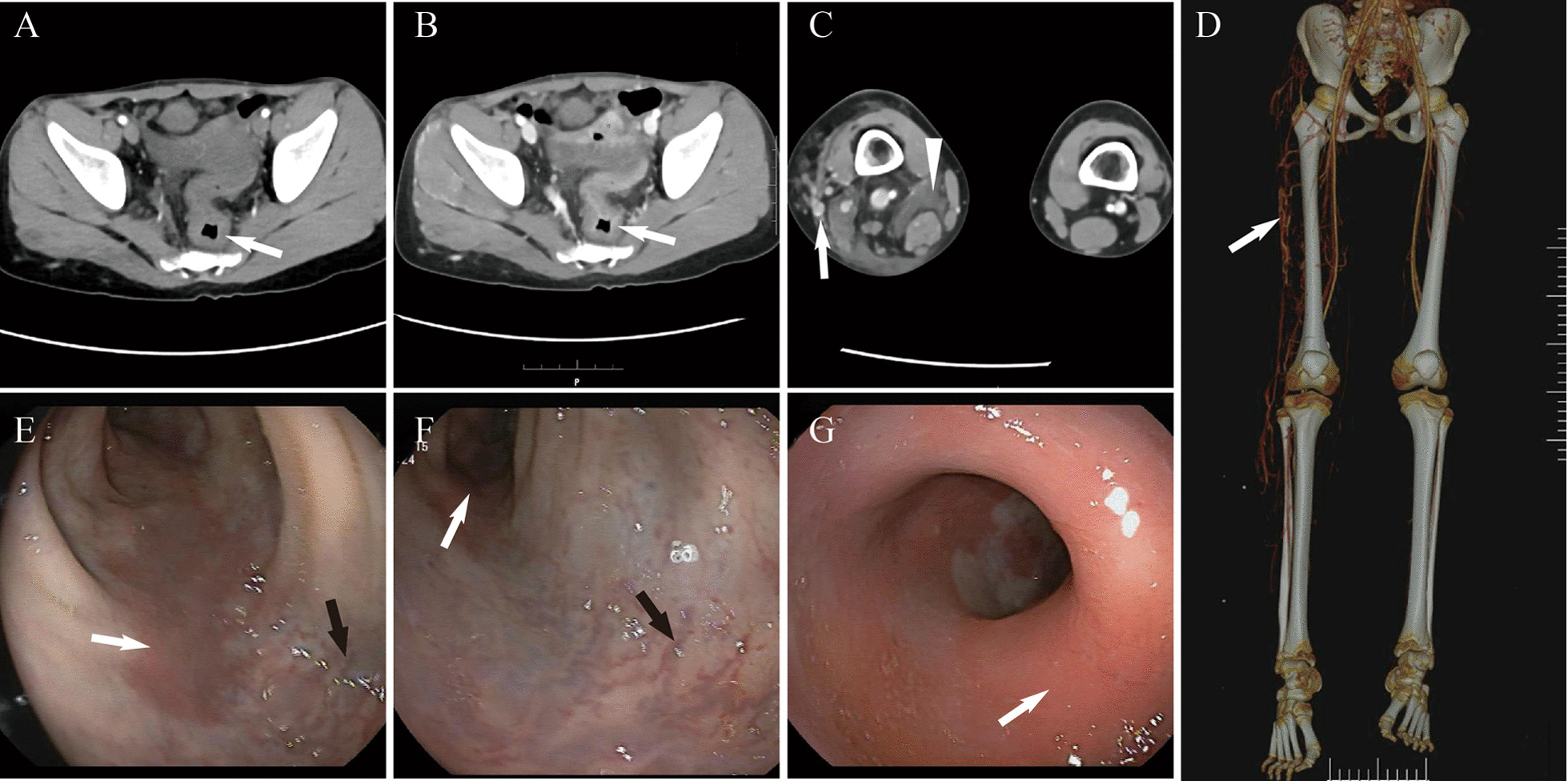
Pelvic and lower limb CT and endoscopy of sigmoid colon and rectum. **A** CT of the sigmoid colon showing thickening of the intestinal wall (arrow). **B** CT showing progressive enhancement of the thickened wall (arrow). **C** CT of the right lower limb showing hypertrophy of the soft tissue, varices (arrow), lymphatic malformations and lymphedema (arrowhead). **D** Volume rendering showing limb-length asymmetry and varices (arrow). **E**–**G** Colonoscopy images showing scattered and continuous erythema (white arrows). **E**, **F** Colonoscopy images revealing dilated vessels (black arrow)

Pelvic CT showed concentric thickening of the sigmoid colon and rectum, with a thickness of 7.8 mm (Fig. 1A). The thickened intestinal wall showed progressive enhancement after the administration of a contrast agent (Fig. 1B). Axial CT images and volume rendering also showed bone and soft tissue hypertrophy of the right lower limb (Fig. 1C, D). Multiple tortuous varices, scattered cystic lymphatic malformations and lymphedema were noted in the fat and muscle layer in the right hip and right lower limb (Fig. 1C). Varices were mainly located on the lateral aspect of her right lower limb (Fig. 1D).

Colonoscopy revealed patchy, branch-like and erythematous almost submucosal appearing lesions affecting the distal sigmoid colon and rectum (Fig. 1E–G), and tortuous and dilated blood vessels were also observed (Fig. 1E, F). Lesions in the distal rectum had surrounded the intestinal wall (Fig. 1G). No active bleeding was found when endoscopy was performed. Colon polyps and colitis were excluded. Vascular malformations of the sigmoid colon and rectum were confirmed using endoscopy. Finally, the patient was diagnosed with KTS with gastrointestinal tract involvement. The patient improved after receiving hemostatic treatment with intravenous injection of hemocoagulase (Hemocoagulase Injection, Slounas, Zhaoke, Hefei) at 0.4 u twice a day, intravenous drip of carbazochrome sodium sulfonate (Carbazochrome Sodium Sulfonate for Injection, Welman, Anhui) at 20 mg once a day for 6 days, and iron supplements for her self-limiting bleeding. She was discharged and followed-up. The patient had a small amount of blood in her stool due to the occasional hard stool. Her hemoglobin remained at approximately 11.0 g/dl (normal range; 11.0–15.0 g/dL) without blood transfusion over 21 months of follow-up.

## Discussion and conclusions

KTS is a disorder combining vascular malformations and an affected overgrowth of a limb. It is believed to be caused by somatic mosaicism in the phosphoinositide 3-kinase/PIK3CA signaling pathway [3], with an incidence of 1 per 100,000 [4]. Common clinical presentations include limb hypertrophy (limb-length asymmetry and soft tissue hypertrophy), varices and vascular nevus, commonly termed a classic triad. KTS diagnosis depends on physical and imaging examinations. Other site involvement causing compression and bleeding has also been reported, and include; the central nervous system [5], the gastrointestinal tract [6], the uterus [7] and the urinary bladder [8].

Gastrointestinal tract involvement accounts for approximately 20% of KTS cases [9]. The sigmoid colon and rectum are the most commonly affected areas. Clinical manifestations range from symptom-free to life-threatening gastrointestinal bleeding [6, 10]. Thus, condition awareness is paramount for appropriate clinical management. In terms of treatment, conservative approaches can be used for patients with self-limiting bleeding. Endoscopic treatments such as endoscopic laser therapy and endoscopic clipping [11] can be applied for localized lesions [9], whereas surgical resection such as sphincter-preserving excision of the affected sigmoid colon and rectum followed by a colon pouch anal anastomosis and protective loop ileostomy may be used for patients with more extensive lesions and severe symptoms [10, 12]. As a preoperative management for surgical resection, selective feeding vessels angiographic embolization can help reduce intraoperative bleeding [2].

In conclusion, gastrointestinal tract involvement should be considered an alternative etiology for unexplained gastrointestinal bleeding in patients with KTS. Imaging provides a non-invasive modality to identify lesion extent, especially if these lesions are located in the small intestine. Critically, using CT and contrast agents, marked thickening of the affected bowel and progressive enhancement can be observed. Endoscopy facilitates direct observation of the mucosa, helps determine the nature of the lesion and identifies the bleeding site. Thus, the synergistic combination of imaging examinations and endoscopy can help diagnose this condition. Treatment modalities depend on the extent of lesions, and patient symptoms.

## Data Availability

All available data are presented in the case.

## Abbreviations

KTS: Klippel–Trenaunay syndrome
CT: Computed tomography
PIK3CA: Phos-phatidylinositol-4,5-bisphospate 3-kinase, catalytic subunit α
